# Propensity matched comparison of omaveloxolone treatment to Friedreich ataxia natural history data

**DOI:** 10.1002/acn3.51897

**Published:** 2023-09-10

**Authors:** David R. Lynch, Angie Goldsberry, Christian Rummey, Jennifer Farmer, Sylvia Boesch, Martin B. Delatycki, Paola Giunti, J. Chad Hoyle, Caterina Mariotti, Katherine D. Mathews, Wolfgang Nachbauer, Susan Perlman, S.H. Subramony, George Wilmot, Theresa Zesiewicz, Lisa Weissfeld, Colin Meyer

**Affiliations:** ^1^ Departments of Pediatrics and Neurology The Children's Hospital of Philadelphia Philadelphia Pennsylvania USA; ^2^ Perelman School of Medicine University of Pennsylvania Philadelphia Pennsylvania USA; ^3^ Reata Pharmaceuticals Dallas Texas USA; ^4^ Clinical Data Science GmbH Basel Switzerland; ^5^ Friedreich Ataxia Research Alliance Downingtown Pennsylvania USA; ^6^ Department of Neurology Medical University Innsbruck Innsbruck Austria; ^7^ Victorian Clinical Genetics Services Murdoch Children's Research Institute Parkville Victoria Australia; ^8^ University College London Hospital Bloomsbury London UK; ^9^ Department of Neurology Ohio State University College of Medicine Columbus Ohio USA; ^10^ IRCCS – Istituto Neurologico Carlo Besta Milan Lombardy Italy; ^11^ Department of Pediatrics University of Iowa Carver College of Medicine Iowa City Iowa USA; ^12^ Department of Neurology University of California Los Angeles Los Angeles California USA; ^13^ Department of Neurology, McKnight Brain Institute University of Florida Health System Gainesville Florida USA; ^14^ Department of Neurology Emory University School of Medicine Atlanta Georgia USA; ^15^ Department of Neurology University of South Florida Ataxia Research Center Tampa Florida USA; ^16^ WCG‐Statistics Collaborative Washington DC USA

## Abstract

**Objective:**

The natural history of Friedreich ataxia is being investigated in a multi‐center longitudinal study designated the Friedreich ataxia Clinical Outcome Measures Study (FACOMS). To understand the utility of this study in analysis of clinical trials, we performed a propensity‐matched comparison of data from the open‐label MOXIe extension (omaveloxolone) to that from FACOMS.

**Methods:**

MOXIe extension patients were matched to FACOMS patients using logistic regression to estimate propensity scores based on multiple covariates: sex, baseline age, age of onset, baseline modified Friedreich Ataxia Rating scale (mFARS) score, and baseline gait score. The change from baseline in mFARS at Year 3 for the MOXIe extension patients compared to the matched FACOMS patients was analyzed as the primary efficacy endpoint using mixed model repeated measures analysis.

**Results:**

Data from the MOXIe extension show that omaveloxolone provided persistent benefit over 3 years when compared to an untreated, matched cohort from FACOMS. At each year, in all analysis populations, patients in the MOXIe extension experienced a smaller change from baseline in mFARS score than matched FACOMS patients. In the primary pooled population (136 patients in each group) by Year 3, patients in the FACOMS matched set progressed 6.6 points whereas patients treated with omaveloxolone in MOXIe extension progressed 3 points (difference = −3.6; nominal *p* value = 0.0001).

**Interpretation:**

These results suggest a meaningful slowing of Friedreich ataxia progression with omaveloxolone, and consequently detail how propensity‐matched analysis may contribute to understanding of effects of therapeutic agents. This demonstrates the direct value of natural history studies in clinical trial evaluations.

## Introduction

Friedreich ataxia (FRDA) is an autosomal recessive neurodegenerative disorder resulting from deficiency of the protein frataxin.^1^, ^2^, ^3^ This deficiency leads to decreased adenosine triphosphate (ATP) production, abnormalities of oxidative phosphorylation, and a diminished antioxidant response.^1^, ^2^, ^3^, ^4^, ^5^, ^6^ Suppression of activity of the transcription factor nuclear factor erythroid‐derived 2‐related factor 2 (Nrf2), which has been documented in FRDA patients and animal models, contributes to impairment of mitochondrial energy production and oxidative damage.^7^, ^8^, ^9^, ^10^ These cellular events lead to the clinical phenotype of progressive ataxia, dysarthria, sensory loss, dyscoordination, and cardiomyopathy. While neurological dysfunction is present in all subjects, the speed of progression varies based on genetic severity and other factors.^11^, ^12^, ^13^

The clinical features of FRDA have become better understood through large ongoing natural history studies. Beginning in 2003, the Friedreich Ataxia Clinical Outcome Measures Study (FACOMS) follows more than 1250 participants at 14 participating clinical sites.^12^, ^13^, ^14^, ^15^ It (along with a parallel study called the European Friedreich's Ataxia Consortium for Translational Studies, EFACTS)^11^ has mapped the disease course, helped develop outcome measures and biomarkers, generated more than 30 scientific publications, and established the infrastructure for conducting more than 10 FRDA clinical trials.^16^, ^17^ FACOMS has focused on measuring the neurological symptoms of FRDA, as well as cardiac disease, vision changes, scoliosis, diabetes and other medical conditions, and medications. The study has led to the development and validation of the Friedreich Ataxia Rating Scale (FARS), a scale including neurologic signs and functional assessments reflecting specific neural features of FRDA.^14^, ^15^, ^18^ Removing items of limited functional significance (such as peripheral nerve elements) improved the measure, creating the modified FARS (mFARS), now used as the primary outcome measure in multiple clinical trials including MOXIe, a randomized, double‐blind trial in FRDA^19^, ^20^ with an ongoing extension.

In addition to characterizing the disease and developing outcome measures, natural history studies can provide control populations for other studies, including intervention studies. This is important when a true placebo is not available (surgical interventions), or study subjects are difficult to recruit (rare disease). Although clinical trials and natural history studies do not provide identical study populations, several approaches can minimize bias and allow a systematic assessment of therapeutic response. The size of the FACOMS database makes a propensity score analysis with optimal 1:1 matching strategy feasible. Therefore, to determine the suitability of the FACOMS dataset as a source of relevant external control data for omaveloxolone in the MOXIe extension study, we performed a propensity‐matched analysis between MOXIe extension data and FACOMS natural history data to assess the value of this natural history study in understanding the effects of omaveloxolone in FRDA.

## Methods

### FACOMS

The studies were approved by the IRB at each institution (primary site, the Children's Hospital of Philadelphia, #11262). Written informed consent was required. FACOMS (NCT03090789) is continuously enrolling with more than 1,000 patients to date.^13^, ^14^, ^15^, ^16^, ^17^ Patients are evaluated annually on FARS/mFARS, other neurologic outcomes, and quality‐of‐life assessments. All sites receive training on the protocol, procedures, and data entry into standardized case report forms. Study investigators meet every 3–6 months to review study conduct, data analysis, results, publications, and study‐related issues.

### MOXIe study extension

The time period for FACOMS overlaps with the MOXIe study (NCT02255435), which consists of three parts (Part 1, Part 2, and extension); the first patient enrolled in Part 2 in October 2017, the last visit occurred in October 2019, and the extension study remains ongoing. All subjects provided written informed consent, and the study was approved by local Institutional Review Boards (IRBs). The ongoing MOXIe extension assesses long‐term safety and tolerability of omaveloxolone in patients with FRDA who completed MOXIe Part 1 or Part 2, both of which were placebo‐controlled.^19^, ^20^ Patients and investigators remain blinded to their preceding study treatment throughout the extension. All patients in the extension receive open‐label omaveloxolone (150 mg) once daily. Efficacy assessments including the mFARS are conducted at baseline (extension Day 1) and then every 24 weeks.

### Primary endpoint

The mFARS is a zero‐ to 99‐point scale comprised of subsections A, B, C, and E of the FARS. A higher score signifies more impairment, and a reduction in score signifies improvement.^15^, ^21^ For the present study, the primary efficacy endpoint was the change in mFARS score from baseline at Year 3 while secondary endpoints were the change in mFARS score from baseline at Years 1 and 2.

### Statistical plan

The methodology for these post hoc analyses, including the determination of covariates used for propensity scores for matching, was developed with several FRDA experts, including the lead author (DRL), the FACOMS statistician (CR), representatives from Friedreich Ataxia Research Alliance (FARA) [JF], statisticians at Reata (AG), and external statisticians from WCG‐Statistics Collaborative (LW). This study uses data from a 24 March 2022 interim database lock for the MOXIe extension. The FACOMS data were current as of 24 March 2021 when obtained from the Critical Path Institute (https://c‐path.org/programs/dcc/projects/friedreichs‐ataxia/).

**Figure 1:**
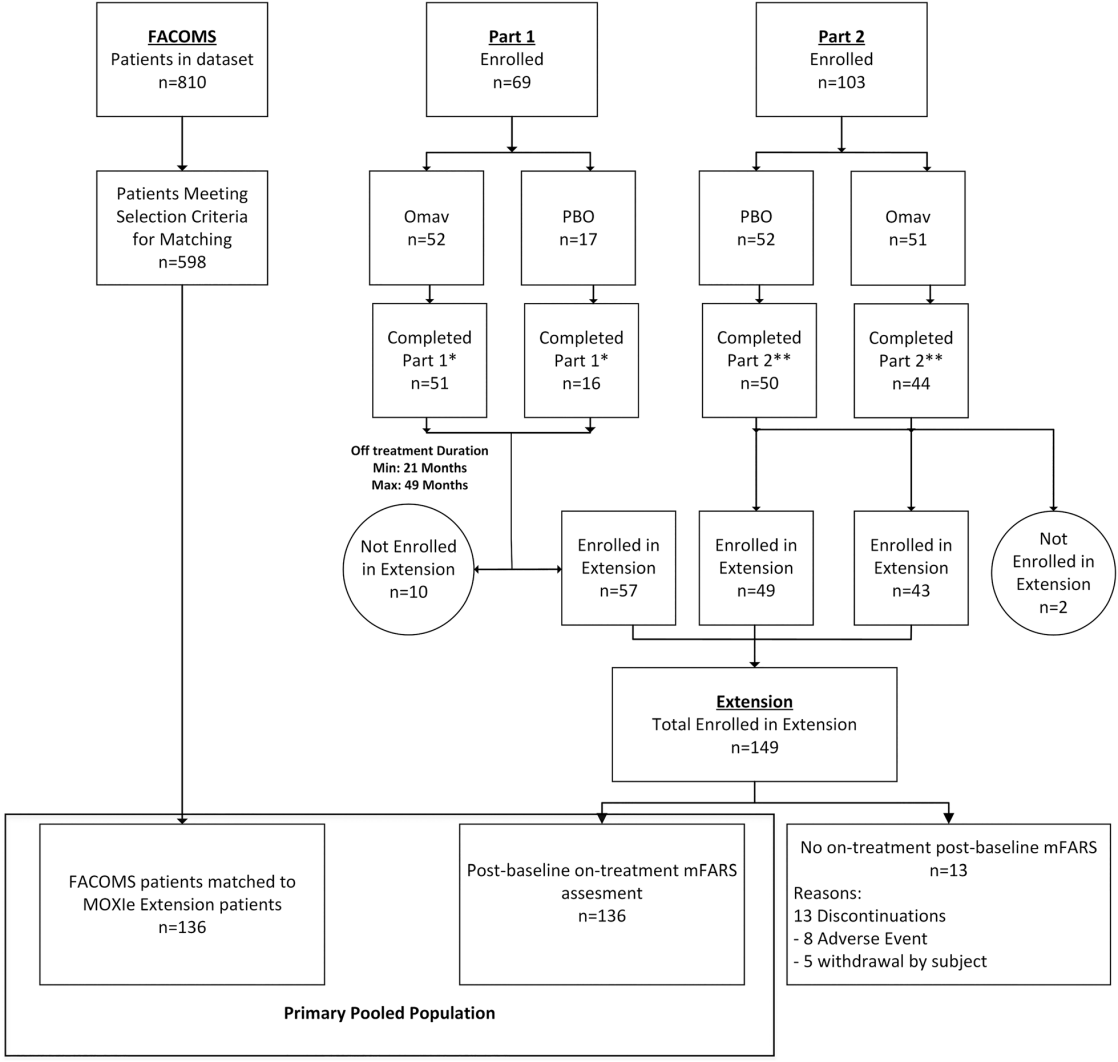
CONSORT diagram of entire study. Omav, omaveloxolone; PBO, placebo.*Completed 12 weeks of treatment. **Completed Week 48 on treatment and had a Week 52 visit.

**Figure 2:**
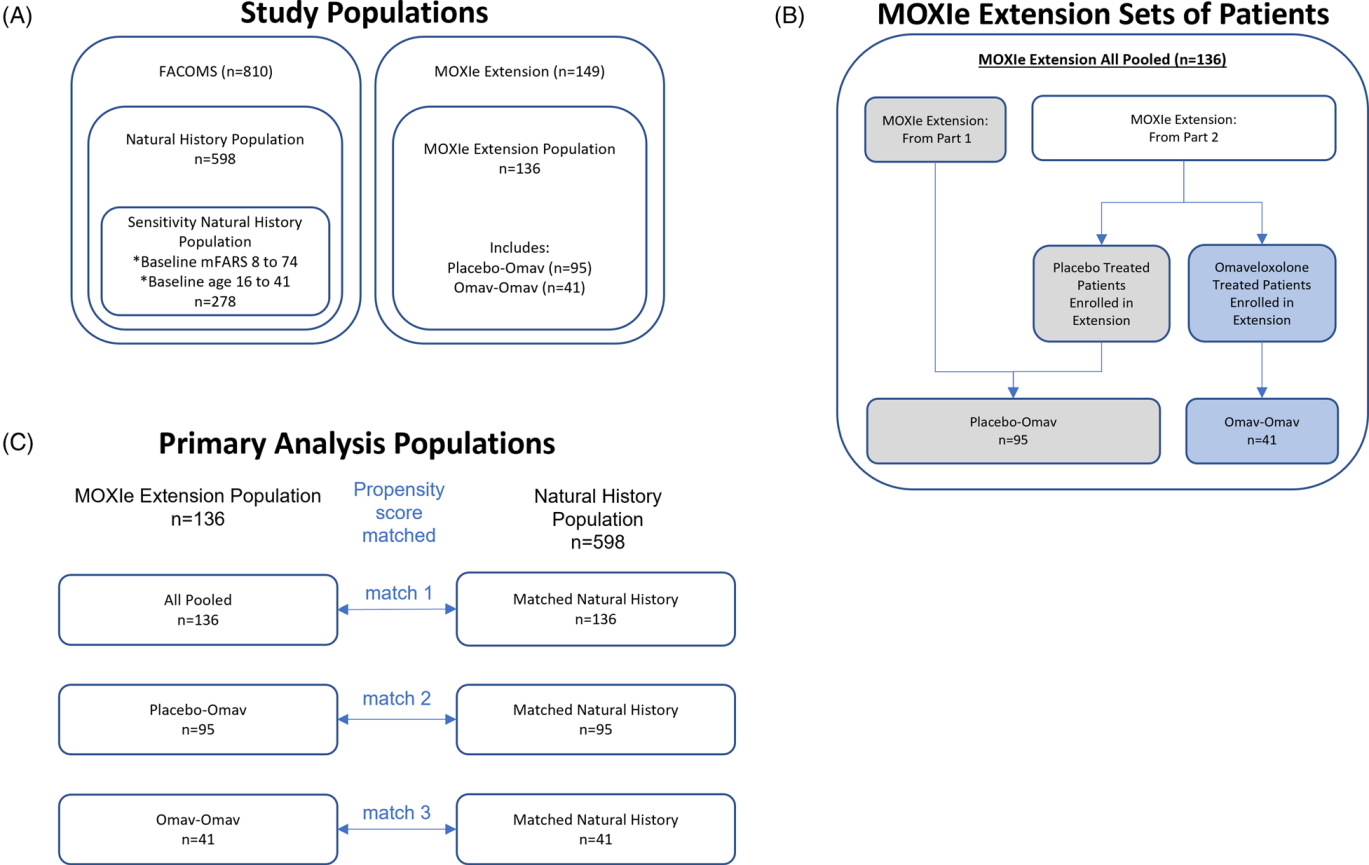
Diagram of study populations (A), MOXIe extension sets (B), and matching process (C). (A) Study populations. Populations were selected from the FACOMS and MOXIe extension studies. All study populations require a baseline mFARS, at least one post‐baseline mFARS within 3 years after baseline, and values for all propensity score model covariates. For the sensitivity natural history population, baseline mFARS and age were within the range observed in MOXIe extension patients at baseline. (B) MOXIe extension sets of patients. From the extension participants, subjects were further classified as to whether they were on sustained omaveloxolone before starting the extension. (C) Primary analysis populations. The propensity matching process was carried out separately for the three analysis sets.

In the analysis, data were compared between MOXIe extension patients (treatment) and matched FACOMS natural history patients (external control) (Fig. 1). The study populations were derived from the full MOXIe extension dataset (*n* = 149) and the full FACOMS dataset (*n* = 810) (Fig. 2A). For inclusion in each study population, patients must have had a baseline mFARS, at least one post‐baseline mFARS within 3 years after baseline, and values for all propensity score model covariates (sex, baseline mFARS score, age at baseline, age of FRDA onset, baseline gait score). All patients were included, regardless of pes cavus status. Three study populations were defined: the MOXIe extension population, the natural history (NH) population, and the sensitivity NH population (SNH). The SNH was defined as the subset of patients from the NH population who had a baseline mFARS score within the range observed at baseline in the MOXIe extension (mFARS 8 to 74) and an age at baseline within the MOXIe extension baseline range (16–41 years). These were chosen as additional criteria because, on a population level, age is the best predictor of mFARS progression rate,^13^ and baseline mFARS score best controls for ceiling effects in the mFARS scale.^14^

### Analysis populations

The MOXIe extension population (Fig. 2B) can be divided into two sets of patients based on prior treatment status: (1) patients considered treatment‐naïve, and (2) patients continuing treatment from their prior study. The first patient was enrolled in the extension after Part 2 recruitment was complete. For MOXIe Part 1 patients, this resulted in a minimum 21‐month off‐treatment period prior to enrolling in the MOXIe extension (Fig. 1). Due to this long off‐treatment period and the short treatment duration in Part 1 (12 weeks), patients from Part 1 were considered treatment‐naive upon entry into the extension and included in the placebo‐omaveloxolone (placebo‐Omav) group. Patients who received placebo in Part 2 were also considered treatment‐naive and included in the placebo‐Omav group. Only those patients who received omaveloxolone in Part 2 and continued treatment in the extension were in the omaveloxolone‐omaveloxolone (Omav‐Omav) group.

Each analysis population was based on a new propensity score match. The primary analysis populations (primary pooled, primary placebo‐Omav, primary Omav‐Omav) were based on matches with the NH population (Fig. 2C). The sensitivity analysis populations (sensitivity pooled, sensitivity placebo‐Omav, sensitivity Omav‐Omav) were based on matches with the SNH population.

### Propensity score matching

Propensity score matching creates comparable groups through the estimation of the propensity score, defined as the probability that a patient received omaveloxolone (i.e., enrolled in MOXIe extension) given a set of covariates. Propensity score matching mimics some characteristics of a randomized study. The observed covariates used for determining propensity scores are controlled for in patients having the same propensity score. Therefore, differences between the MOXIe extension and FACOMS groups should be accounted for and are likely not a result of observed covariates.

The propensity score is a linear combination of covariates requiring that patients have a similar propensity score rather than a caliper match on a group of covariates. Computation of the propensity score was coupled with diagnostics to assess the adequacy of matching techniques used in the analysis. The matching was carried out as optimal 1:1 matching without replacement.

Several assumptions were made when creating the analysis populations for the proposed design and propensity score computation. These assumptions include

- Strongly ignorable treatment assignment:^22^ The treatment assignment must be independent of the change from baseline in mFARS score over time given the covariates used in the analysis. There is a positive probability of being in the omaveloxolone or the FACOMS population, that is the propensity score estimated from the logistic regression model must be strictly greater than 0 and less than 1.
- Stable‐unit treatment value assumption:^23^, ^24^ The outcomes of one individual are not affected by the group assignment of another.

These assumptions were met in this approach using propensity scores.

Computation of the propensity score. The propensity score was estimated using logistic regression with covariates. The criteria for determining model fit differ from those for standard logistic regression analysis, as the goal of propensity score analysis is to balance key covariates across the MOXIe extension patients and control patients, not to estimate a treatment effect. Omission of covariates potentially related to the outcome could increase bias, arguing for a strategy of including more, rather than fewer covariates in the model.

Factors established as prognostic and available in both FACOMS and MOXIe studies were selected as covariates for the logistic regression model used for determining propensity scores (Table 1).^21^, ^25^ Some factors, such as the guanine‐adenine‐adenine 1 (GAA1) repeat length (the shorter of the two *FXN* intron 1 GAA repeats), may be prognostic but were not available for all patients. Notably, the presence of pes cavus was not a matching criterion for the FACOMS external cohort as it was not systematically evaluated or available for all patients.

**Table 1:** Covariates used in propensity score matching.

Covariate	Rationale	Reference	Number (%) of FACOMS patients with data (*n* = 810)	Number (%) of MOXIe extension patients with data (*n* = 149)
Age	Primary determinant of severity	Patel et al.^13^	807 (99.6%)	149 (100%)
Age of onset	Surrogate for rate of progression and GAA repeat length	Patel et al.^13^	801 (98.9%)	149 (100%)
Sex	Sexual dimorphisms observed in ataxia studies	Klockgether et al.^25^; Friedman et al.^21^	810 (100%)	149 (100%)
Baseline gait score	Allows matching of patients at same functional level	Rummey et al.^12^	790 (97.5%)	149 (100%)
mFARS score at baseline	Allows matching of patients at same functional level	Rummey et al.^12^	789 (97.4%)	149 (100%)
Other covariates considered but not included				
GAA1 repeat length	Not included		745 (92.0%)	131 (87.9%)
Pes cavus	Not included		432 (53.3%)	149 (100%)

List of covariates for propensity matching. The definition of pes cavus between the two studies was not consistent. Pes cavus was based on clinical judgment in FACOMS but by detailed testing in the MOXIe extension.

FRDA, Friedreich ataxia; mFARS, Modified Friedreich ataxia rating scale.

Creation of the analysis populations from the propensity analysis. After computation of the initial propensity score for males and females, optimal matching was used to create the matched population that was used for the analysis. Diagnostics were then used to assess the similarity of the two groups and whether the propensity score model was adequately specified.

### Efficacy analysis considerations

#### Definition of baseline

Baseline values were defined as the last non‐missing assessment prior to the first study drug administration in MOXIe extension. For FACOMS patients, baseline was defined as having a record marked VISIT = Day 1. As an example, if a patient had no recorded FARS assessment at the baseline visit, but they did have a FARS assessment at Year 2, the Year 2 assessment was not considered as baseline and the patient was not included in the analysis as they did not have a baseline assessment.

#### Primary efficacy analyses

The change from baseline in mFARS at Year 3 was analyzed using a mixed model repeated measures (MMRM) model that included treatment group, baseline mFARS, visit, and interaction terms for visit‐by‐baseline and treatment group‐by‐visit as covariates. The model was fit using restricted maximum likelihood with a Toeplitz covariance structure (assuming measurements taken closer together in time are more highly correlated than those taken farther apart). Least squares (LS) mean changes from baseline, between group differences in LS mean changes from baseline and associated p‐values estimated from the MMRM model are reported.

*Visit schedule*. Annual visits for the MMRM analysis were defined to align with the FACOMS assessment schedule. The mFARS assessment collected closest to 1, 2, and 3 years after baseline was used for each of the annual assessments.

#### Secondary and sensitivity analyses

The secondary endpoints were analyzed using the same MMRM model as for the primary outcome of change from baseline in mFARS at Year 3. For sensitivity analyses, the analyses above were repeated for each sensitivity population.

Statistical analyses were conducted with SAS software version 9.4 (TS1M6). Further statistical details are included in a Supplemental Statistical Appendix.

## Results

### Patients

Overall, 149 patients were enrolled in the MOXIe extension, 57 patients from Part 1 and 92 from Part 2 (Fig. 1). Of these, 136 had a post‐baseline mFARS assessment while on treatment and were included in the propensity‐matched analysis for the primary pooled population; the other 13 were excluded from further study. For the 136 patients in the primary pooled population, the median treatment duration in MOXIe extension (exclusive of treatment duration in Part 1 or Part 2) was 2.76 years, with a maximum of 3.4 years and a minimum of 0.5 years as of 24 March 2022 (Table 2).

**Table 2:** Follow‐up/exposure duration (primary pooled population).

Statistic	Matched FACOMS follow‐up (*N* = 136)	MOXIe extension exposure (*N* = 136)
	Years	Years
Mean (SD)	2.54 (0.786)	2.60 (0.524)
Median	2.92	2.76
Min, max	0.6, 3.5	0.5, 3.4

Follow‐up duration in the natural history study is reported for the matched FACOMS patients, and exposure to omaveloxolone is reported for MOXIe extension patients.

Max, maximum; Min, minimum; SD, standard deviation.

The FACOMS dataset received from C‐Path included 810 patients who consented to have their data shared outside of the core FACOMS study. Of these, 598 met the criteria for inclusion in the NH study population, and 278 patients met criteria for inclusion in the SNH study population. These were included as potential matches to patients in the MOXIe extension. The FACOMS external cohort that was the matched set for the MOXIe extension patients in the primary pooled population consisted of 136 patients. These patients had a median follow‐up duration in the ongoing FACOMS natural history study of 2.92 years, with a maximum of 3.5 years and a minimum of 0.6 years (Table 2). In total, the primary pooled population included 272 patients (136 from the MOXIe extension and 136 matched patients from FACOMS).

### Demographics and baseline characteristics

Covariates used for determining the propensity scores were balanced between the groups (Table 3) as were other demographics and baseline characteristics (Table S1). Slight differences observed in GAA1 and GAA2 (the longer of the two FXN GAA intron 1 repeats) repeat length, although significant, were not clinically meaningful based on ceiling effects of the GAA1 length.^26^, ^27^, ^28^ Eligible patients from FACOMS who were not matched in the primary pooled population had similar baseline characteristics to those who were matched (Table S2).

**Table 3:** Demographics and baseline characteristics used as covariates for propensity score calculation (primary pooled population).

Characteristic	Statistic	Matched FACOMS	MOXIe extension
Age (years)	*n*	136	136
	Mean (SD)	26.2 (13.7)	26.6 (7.3)
	Min, max	6, 64	16, 41
	*p* value	–	0.76
Age at FRDA onset	*n*	136	136
	Mean (SD)	15.2 (10.5)	15.5 (5.3)
	*p* value	–	0.81
Sex (*n* [%])	*n*	136	136
	Female	70 (51.5%)	70 (51.5%)
	Male	66 (48.5%)	66 (48.5%)
	*p* value	–	1
mFARS	*n*	136	136
	Mean (SD)	41.0 (16.1)	42.2 (12.6)
	Min, max	5.3, 77.0	8.2, 73.5
	*p* value	–	0.50
Gait (assessment #7 in FARS section E [upright stability])	*n*	136	136
	Mean (SD)	2.7 (1.69)	2.8 (1.36)
	*p* value	–	0.58

*p* value for the difference between MOXIe extension and matched FACOMS was obtained by two‐sample *t* test for age, age at FRDA onset, mFARS and gait and by chi‐square test for sex.

FRDA, Friedreich ataxia; FARS, Friedreich ataxia rating scale; Max, maximum; mFARS, modified Friedreich ataxia rating scale; Min, minimum; SD, standard deviation.

Demographics and baseline characteristics for the primary placebo‐Omav and primary Omav‐Omav populations were generally well‐balanced between the groups in both populations, although differences as in the primary pooled population in GAA1 and GAA2 repeat length were observed (Tables S3 and S4 respectively). Similarly, demographics and baseline characteristics were well‐balanced in the sensitivity populations (Tables S5–S7).

Across analysis populations, there were no significant differences in the characteristics used as covariates for matching. Differences in other baseline characteristics were not considered clinically meaningful, and significant differences were infrequently observed in height, weight and select vital sign assessments across the analysis populations.

### Propensity of matching

For the propensity score model used in this analysis, the diagnostics used to assess comparability of the FACOMS and MOXIe extension subjects indicated that the quality of the matches was good for all populations (Table 4; Table S8). There were some instances where diagnostics fell below the “acceptable” range^29^ (PROC PSMATCH documentation) for the comparison of the variances of the residuals for covariates in the two groups. This was true for the ratio of the variances of the residuals for age and age of onset covariates which were more variable than other covariates in the model (Table 3). Taken together, the diagnostic results show a high quality of matching for the primary pooled population, primary placebo‐Omav population, and primary Omav‐Omav population.

**Table 4:** Propensity score diagnostic results (primary populations).

Diagnostic	Criteria for good or acceptable match^a^	Pooled (Match 1)	Placebo‐Omav (Match 2)	Omav‐Omav (Match 3)
		Score	Score	Score
Standardized difference of the means of the propensity score	<0.5	0.0055	0.0090	0.0012
Standardized difference of the means of covariates				
Sex	<0.5	0	0	0
Baseline gait	<0.5	0.0672	0.0802	0.0325
Baseline mFARS	<0.5	0.0826	0.1103	0.0828
Age at baseline	<0.5	0.0375	0.0902	0.1357
Age at FRDA onset	<0.5	0.0292	0.0645	0.0424
Ratio of the variances of the propensity score	Close to 1; >0.8 and <1.25	1.02	1.04	0.997
Ratio of the variances of the residuals for covariates				
Sex	0.5 to 2	1.00	1.00	0.999
Baseline gait	0.5 to 2	0.575	0.502	0.560
Baseline mFARS	0.5 to 2	0.607	0.499	0.548
Age at baseline	0.5 to 2	0.343	0.331	0.201
Age at FRDA onset	0.5 to 2	0.319	0.285	0.433

^a^ Criteria for a “good” match shown for standardized difference of the means of the propensity score, standardized difference of the means of the propensity score for each covariate, and ratio of the variances of the propensity score. Criteria for an “acceptable” match shown for the ratio of the variances of the residuals for each covariate.

FRDA, Friedreich ataxia; mFARS, modified Friedreich ataxia rating scale.

Efficacy Results. Results for the primary endpoint, change from baseline in mFARS score at Year 3, differed significantly between patients receiving omaveloxolone in MOXIe extension and the untreated matched FACOMS patients. After 3 years, in the Pooled Primary Population, matched FACOMS patients progressed 6.6 mFARS points whereas patients treated with omaveloxolone in MOXIe extension progressed 3.0 points (difference = −3.6 points; nominal *p* = 0.0001) (Table 5); thus, progression in mFARS was reduced by 55% in the omaveloxolone treatment group compared to the matched group. Analysis of the primary endpoint in the primary placebo‐Omav and primary Omav‐Omav populations yielded similar results, with nominal p‐values of <0.05 for the treatment difference and slowing of progression in mFARS of >50% (Table 6). In the primary placebo‐Omav population, containing treatment‐naive patients at extension baseline, progression in mFARS was slowed by 56% compared to NH controls. In the Primary Omav‐Omav Population, in which extension patients had previously received 48 weeks of omaveloxolone treatment in Part 2, progression in mFARS was slowed by 61% in MOXIe extension patients compared to FACOMS patients, suggesting that such patients continue to benefit from omaveloxolone treatment (Table 6).

**Table 5:** Analysis of primary outcome measure.

	Pooled (Match 1)	
	Matched FACOMS (*N* = 136)	MOXIe extension (*N* = 136)
Baseline, mean (±SD)	41.0 (16.1)	42.2 (12.6)
mFARS change from baseline (LS mean [±SE])		
Year 3	6.61 (0.65)	3.00 (0.66)
Year 3 difference		−3.61 (0.93) *p* = 0.0001

Difference is MOXIe extension—matched FACOMS.

LS, least squares; mFARS, modified Friedreich ataxia rating scale; SD, standard deviation; SE, standard error.

**Table 6:** Analysis of primary outcome measure in additional analysis populations.

	Placebo‐Omav (Match 2)		Omav‐Omav (Match 3)	
	Matched FACOMS (*N* = 95)	MOXIe extension (*N* = 95)	Matched FACOMS (*N* = 41)	MOXIe extension (*N* = 41)
Baseline, mean (±SD)	44.5 (18.0)	42.8 (12.8)	39.6 (16.8)	40.9 (12.2)
mFARS change from baseline (LS mean [±SE])				
Year 3	7.29 (0.72)	3.21 (0.76)	6.14 (1.24)	2.38 (1.33)
Year 3 difference		−4.09 (1.05) *p* = 0.0001		−3.76 (1.82) *p* = 0.0400

Difference is MOXIe extension – matched FACOMS.

LS, least squares; mFARS, modified Friedreich ataxia rating scale; SD, standard deviation; SE, standard error.

**Figure 3:**
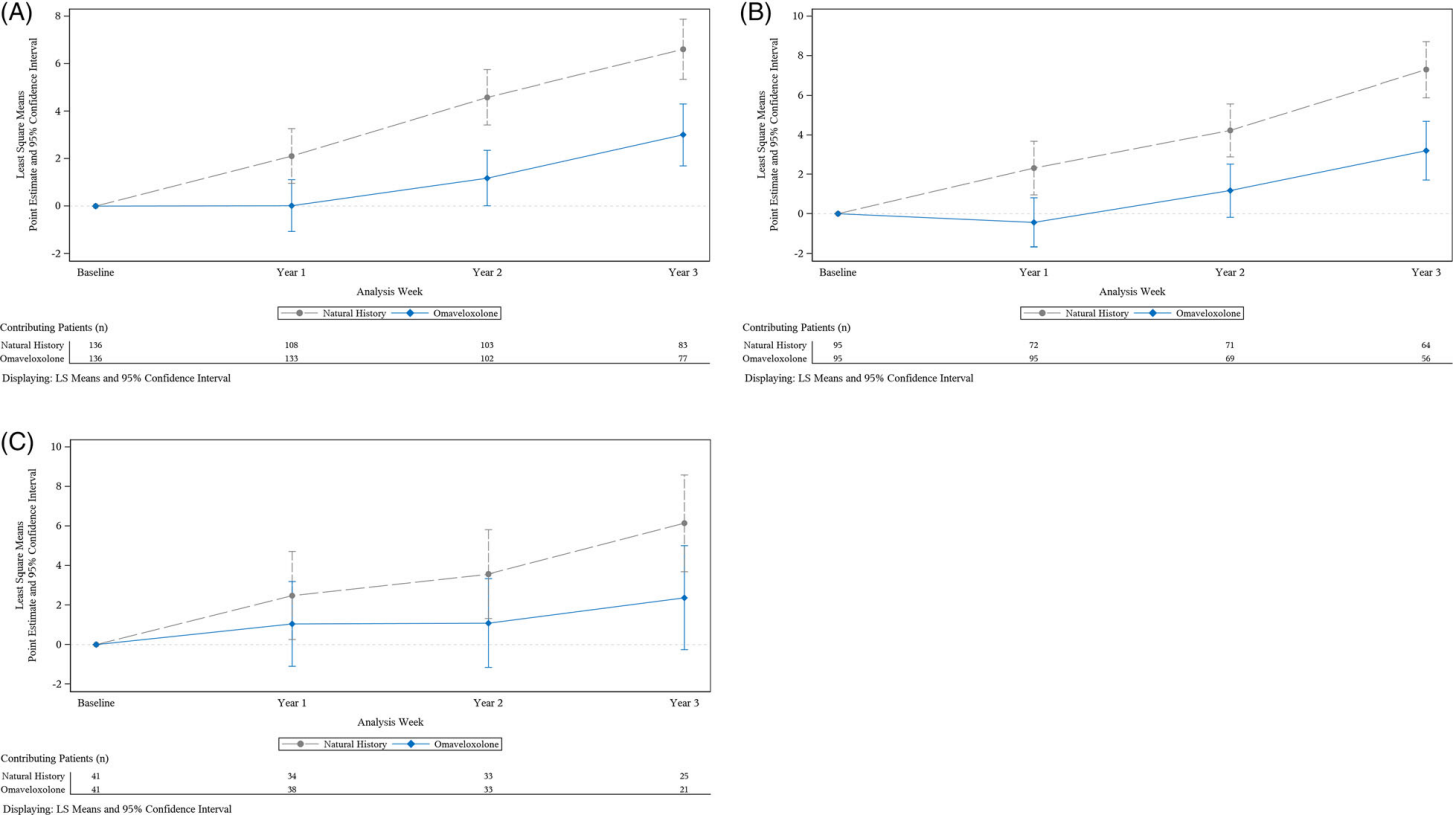
Change in mFARS from baseline over time. (A) Primary pooled population. (B) Primary placebo‐Omav population. (C) Primary Omav‐Omav population.

Results of the secondary endpoints, change from baseline in mFARS score at Year 1 and Year 2, also favor omaveloxolone in the primary pooled population (Table 7; Fig. 3A). At each year, extension patients experienced a smaller change from baseline in mFARS than the matched FACOMS patients. These distinct trajectories over 3 years show consistent separation between the two groups and yield nominal *p*‐values less than 0.05 for all comparisons. The FACOMS patients eligible for matching but who were not matched to MOXIe extension patients in the primary pooled population also showed consistent disease worsening (increase in mFARS) over 3 years (Table S9). In the primary placebo‐Omav population, the treatment effect favored MOXIe extension at Year 1 and Year 2. MOXIe extension patients experienced an improvement (i.e., a decrease) from baseline in mFARS at Year 1, and the treatment difference was −2.8 mFARS points (nominal *p* = 0.0035) (Table 7; Fig. 3B). In the primary Omav‐Omav population, the treatment effect favored the extension at Year 1 and Year 2. A smaller difference between treatment groups was observed at Year 1 in the Omav‐Omav population than in the placebo‐Omav population. Extension patients in the Omav‐Omav population did not experience an improvement from baseline at Year 1, likely because they were in their second year of treatment with active drug. The treatment effect favored the extension at each visit and consistently increased over time in this population (Table 7; Fig. 3C).

**Table 7:** Change in mFARS over 2 years.

	Baseline		mFARS change from baseline			
			Year 1		Year 2	
	*N*	Mean (SD)	*N*	LS mean (±SE)	*N*	LS mean (±SE)
Primary pooled population						
MOXIe extension	136	42.2 (12.6)	133	0.015 (0.56)	102	1.18 (0.59)
Matched FACOMS	136	41.0 (16.1)	108	2.11 (0.59)	103	4.58 (0.59)
Difference	–	–	–	−2.10 (0.81) *p* = 0.010	–	−3.41 (0.84) *p* < 0.0001
Primary placebo‐Omav population						
MOXIe extension	95	42.8 (12.8)	95	−0.43 (0.63)	69	1.18 (0.69)
Matched FACOMS	95	44.5 (18.0)	72	2.32 (0.69)	71	4.23 (0.68)
Difference	–	–	–	−2.75 (0.94) *p* = 0.0035	–	−3.06 (0.97) *p* = 0.0017
Primary Omav‐Omav population						
MOXIe extension	41	40.9 (12.2)	38	1.05 (1.09)	33	1.10 (1.13)
Matched FACOMS	41	39.6 (16.8)	34	2.48 (1.12)	33	3.57 (1.13)
Difference	–	–	–	−1.43 (1.56) *p* = 0.36	–	−2.47 (1.60) *p* = 0.13

Difference is MOXIe extension – matched FACOMS.

LS, least squares; mFARS, modified Friedreich ataxia rating scale; SD, standard deviation; SE, standard error.

### Sensitivity analyses

Results of the primary and secondary endpoints were similar in the sensitivity populations, in which the pool of FACOMS patients available for matching for each extension set was further restricted to patients who had a baseline mFARS value and baseline age within the range observed in the MOXIe extension baseline. At all timepoints, and in all three sensitivity populations (comprised of three separate sets of rematched patients), results favored omaveloxolone (extension patients). At Year 3, the three sensitivity populations showed a treatment benefit of −2.4, −3.2, and −4.7 mFARS points for the sensitivity pooled, sensitivity placebo‐Omav, and sensitivity Omav‐Omav populations, respectively, all with nominal p‐values of <0.05 (Table S10).

## Discussion

The present post hoc results demonstrate that treatment with omaveloxolone provided a clinically meaningful slowing of FRDA progression over a 3‐year period compared to untreated, propensity score‐matched FACOMS external controls. Data from Parts 1 and 2 of MOXIe were not included in this propensity‐matched analysis. After 3 years in the primary pooled population (*n* = 272; 136 from FACOMS and 136 from the MOXIe extension), matched FACOMS patients progressed 6.6 mFARS points whereas patients treated with omaveloxolone in the MOXIe extension progressed 3.0 points (difference = −3.6, nominal *p* = 0.0001); thus, progression in mFARS was slowed by 55% with omaveloxolone relative to natural history controls. Clinical trials in neurodegenerative diseases are typically powered to detect a 50% slowing of progression in 1 year,^21^ showing the context of the potential benefit here relative to other studies. During treatment in the MOXIe extension, omaveloxolone treatment in the primary pooled population slowed progression by >50% at each year compared to the corresponding FACOMS external control group, indicating benefit that persisted and accrued over 3 years. Subsets of MOXIe extension patients that differed in prior treatment status also had slowed disease progression when matched to FACOMS controls. All of the primary analysis populations suggested a benefit of omaveloxolone as did sensitivity analyses at all points during the 3‐year duration of the study. In general, the absolute treatment differences are smaller in the sensitivity populations than in the primary populations. Due to sampling variability a range of responses is expected for these populations; however, despite numerical differences, results favored omaveloxolone in all three primary populations and all three sensitivity populations over 3 years. The percent reduction in disease progression after 3 years of 48% or greater in all sensitivity populations was consistent with the primary populations. In MOXIe Part 2, omaveloxolone‐treated patients experienced a reduction (i.e., an improvement) in mFARS score relative to the placebo group at 48 weeks (−2.4 mFARS points), revealing a benefit of omaveloxolone.^20^ This treatment difference was similar in magnitude to the difference between the MOXIe extension and matched FACOMS patients at Year 1 for the primary placebo‐Omav population (−2.8 mFARS points) in which MOXIe extension patients were considered treatment‐naive at baseline. Together, the present results show a meaningful slowing of FRDA progression with omaveloxolone over a period of 3 years and further support the findings observed in the MOXIe study Part 2.^20^ Thus, the potential benefit of omaveloxolone demonstrated across multiple analysis populations separately matched to FACOMS patients and the comparability of these results to Part 2 results provide supportive evidence for the potential use of omaveloxolone in FRDA.

While there have been many attempts to use natural history studies as controls in clinical trials, the systematic design of such approaches is difficult. Analyses based on external controls are potentially limited by lack of randomization, which can introduce bias; however, International Council for Harmonisation of Technical Requirements for Pharmaceuticals for Human Use (ICH) E10 guidelines advise that bias can be minimized when there is detailed patient information collected and when patients are as similar as possible, including in demographic characteristics and baseline status, between interventional groups and natural history controls. The FACOMS natural history study presents an opportunity for identification of suitable external control groups. The significant overlap in trial sites and investigators (some of whom helped develop FRDA standard of care guidelines) ensures a similar approach to overall patient management for FRDA and comorbid conditions.^30^, ^31^ In addition, at the time of the study, there were no approved or effective disease modifying therapies for FRDA, minimizing confounding in the comparison of groups from two different studies. The overlap in trial sites and the reliance on the same investigator to provide training on mFARS assessments further contributed to consistency in methodology and scoring of mFARS in patients originating from both studies.

Use of propensity score matching provides further rigor in identifying an appropriate external control group aligning with ICH E10 guidance. Using this approach, the matched FACOMS group in the primary pooled population was highly comparable for demographics and baseline characteristics to the MOXIe extension patients, including the characteristics for determining the propensity scores. Similarly, in the sensitivity pooled population, in which the matched set for the analysis was selected from a more restricted pool, demographics and baseline characteristics were highly comparable. Additionally, propensity score matching diagnostic results demonstrated that the propensity score matching was good (i.e., < 0.5) for the standardized difference of the means of the propensity score overall and for each covariate,^24^ good (i.e., close to 1; >0.8 and <1.25) for the ratio of the variances of the propensity score, and acceptable for the ratio of the variances of the residuals for covariates (0.5 to 2)^29^ for most covariates for all analysis populations. Thus, although patients in the analysis come from two different studies with different eligibility criteria, diagnostic results and demographic and baseline characteristic results demonstrate that the MOXIe extension and FACOMS patients were highly comparable. Although the model used for propensity score matching included multiple covariates that were considered prognostic for FRDA progression, the model may have been limited by not also having GAA1 repeat length or pes cavus as covariates. These were not included in the model due to lack of availability of GAA1 data in all patients and differences in the method of evaluation of pes cavus between studies. However, baseline characteristics for mean GAA1 repeat length in both FACOMS and MOXIe extension patients were in a range in which the consequence of longer GAA1 repeat length on disease severity has reached a relative ceiling effect.^25^, ^26^, ^27^ Furthermore, if it were significant, the slightly longer GAA1 length in MOXIe subjects would bias the results toward the null (as longer GAA1 lengths predict faster progression). Pes cavus does not clearly influence the speed of disease progression, and individuals in the double‐blind portion of MOXIe responded to drug, but not as highly as those without pes cavus for reasons that are not entirely understood.

Another potential limitation in the cross‐study comparison was that scheduled assessments for mFARS differed for the two groups, with annual assessments in the FACOMS study and biannual assessments in MOXIe extension. Accordingly, analysis windows were defined using annual visits to align the MOXIe extension schedule with the FACOMS schedule. The MMRM analysis model aligns with the MOXIe Part 2 primary analysis method, is a common model frequently used by the Food and Drug Administration (FDA) and does not rely on a linearity assumption. While the annual visits align with the FACOMS assessment schedule, a limitation of the MMRM analysis using annual visits is not using all mFARS assessments from the MOXIe extension. The mean study day of collection of the mFARS score used for analysis was similar between the treatment groups in all analysis populations at each time point. The biggest difference was observed at Year 3, with mean study day approximately 2 to 3 months later in matched FACOMS patients compared to MOXIe extension for each study population (Table S11); while this may have introduced some bias toward MOXIe extension patients, the 3‐month difference in average time of assessment is unlikely to account for the treatment difference observed at Year 3 in the primary pooled population (−3.6 mFARS points).

In contrast to a placebo‐controlled trial, this analysis using external controls from a natural history study does not account for a placeboeffect. Taken together, a longer duration of follow‐up (3 years) and the magnitude of the treatment effect after 3 years (−3.6 mFARS points in the primary pooled population) reduce the potential for placebo effect impact. Additionally, in this propensity‐matched analysis, the effect size observed at 1 year after baseline in the placebo‐Omav population is similar to that observed in placebo‐controlled MOXIe Part 2. This population is most similar to the MOXIe Part 2 study since patients were considered treatment‐naive upon starting treatment in MOXIe extension. The consistency of treatment effect when using natural history or placebo as the control group suggests minimal placebo effect after Year 1. Thus, the impact of the placebo effect is potentially minor after 3 years in this progressive disease in which patients decline yearly in mFARS score.

In conclusion, although there are limitations to this cross‐study analysis, the approach leads to readily interpretable results on the potential benefit of omaveloxolone. The FACOMS cohort identified by propensity score matching is highly comparable to the MOXIe extension patients for baseline characteristics and standard of care; therefore, the observed difference in disease progression (in mFARS) may be attributed to omaveloxolone treatment. Thus, such propensity‐matched analysis from the FACOMS natural history study compared to MOXIe extension provide evidence for the possible use of omaveloxolone for the treatment of FRDA and demonstrates the value and methodology for utilization of natural history data in clinical trials in rare diseases.

## Author Contributions

DRL, AG, and CM wrote the first draft. All authors provided critical review and feedback and approved the final draft. AG, CR, and LW provided statistical analysis, DRL, JF, AG and CM designed the work. DRL, SB, MBD, PG, JCH, CM, KDM, WN, SP, SHS, GW, and TZ performed examinations and recruited subjects.

## Conflict of Interest

Lynch D receives grants from FARA, the Muscular Dystrophy Association (MDA), the National Institutes of Health (NIH), Reata Pharmaceuticals, and Retrotope. Rummey C reports research and consultancy fees from FARA, The National Ataxia Foundation (NAF), PTC Therapeutics, Reata Pharmaceuticals, Biohaven, Santhera Pharmaceuticals, and Takeda Pharmaceuticals. Boesch S has received fees for consultancy, advisory boards, and/or honoraria from AbbVie, Ipsen, Reata Pharmaceuticals, Merz Pharma, Stada Arzneimittel, and VICO Therapeutics. Delatycki M, Mariotti C, and Mathews K have received funding from Reata Pharmaceuticals during the study. Mathews K reports personal fees for consultancy from VICO Therapeutics; grants from Takeda and FARA. Giunti P has received grants and consultancy fees from Reata Pharmaceuticals, VICO Therapeutics, and consultancy fees from Triplet Therapeutics, and PTC Therapeutics. Nachbauer W reports advisory and speaker honoraria from Reata Pharmaceuticals. Hoyle C has received grants from Reata Pharmaceuticals, Takeda, and FARA during the conduct of the study; personal fees from Reata Pharmaceuticals and Avexis outside the submitted work. Subramony S H reports grants from Reata Pharmaceuticals, Biohaven Pharmaceuticals, Takeda, PTC Therapeutics, Retrotope, Acceleron Pharmaceuticals, NIH, FDA, MDA, Wyck Foundation, FARA, Stealth, Avidity Biosciences, and Fulcrum Therapeutics. Wilmot G has received grant/research support from Reata Pharmaceuticals, Biohaven Pharmaceuticals, NIH, NAF, and FARA. Weissfeld L is an employee of WCG Clinical, Inc. WCG is a paid consultant for Reata Pharmaceuticals. Zesiewicz T has received compensation for serving on the advisory boards of Boston Scientific, Reata Pharmaceuticals, and Steminent Biotherapeutics; has received personal compensation as senior editor for Neurodegenerative Disease Management and as a consultant for Steminent Biotherapeutics; has received royalty payments as co‐inventor of varenicline for treating imbalance (patent number 9,463,190) and non‐ataxic imbalance (patent number 9,782,404); has received research/grant support as principal investigator/investigator for studies from AbbVie; Biogen; Biohaven Pharmaceuticals; Boston Scientific; Bukwang Pharmaceutical Co, Ltd; Cala Health; Cavion; FARA; Houston Methodist Research Institute; NIH (READISCA U01); Retrotope; and Takeda Development Center Americas. Perlman S reports no conflicts or relevant disclosures. Goldsberry A and Meyer C are employees of Reata Pharmaceuticals. Farmer J is an employee of FARA.

## Supporting information


Table S1
