# Supplementary material for: Propensity matched comparison of omaveloxolone treatment to Friedreich ataxia natural history data
Source: Ann Clin Transl Neurol. 2023 Sep 10;11(1):4–16. doi: 10.1002/acn3.51897 (PMC10791025; doi:10.1002/acn3.51897)
Supplement: Supplementary file 1 — Table S1 [file ACN3-11-4-s001.docx]

List of Supplementary Material:

Supplementary Table 1: Other Characteristics (Primary Pooled Population)

Supplementary Table 2: Demographics and Baseline Characteristics (FACOMS Patients Eligible for Matching who Were Not Matched to MOXIe Extension Patients in the Primary Pooled Population)

Supplementary Table 3: Demographics and Baseline Characteristics (Primary Placebo-Omav Population)

Supplementary Table 4: Demographics and Baseline Characteristics (Primary Omav-Omav Population)

Supplementary Table 5: Demographics and Baseline Characteristics (Sensitivity Pooled Population)

Supplementary Table 6: Demographics and Baseline Characteristics (Sensitivity Placebo-Omav Population)

Supplementary Table 7: Demographics and Baseline Characteristics (Sensitivity Omav-Omav Population)

Supplementary Table 8: Propensity Score Diagnostic Results (Sensitivity Populations)

Supplementary Table 9: Change in mFARS over 3 years (FACOMS Patients Eligible for Matching who Were Not Matched to MOXIe Extension Patients in the Primary Pooled Population)

**Supplementary Table 10:** mFARS Change in Sensitivity Populations

Supplementary Table 11: mFARS Study Day at Each Analysis Visit

**Supplementary Statistical Appendix**

Supplementary Table 1: Other Characteristics (Primary Pooled Population)

| Characteristic | Statistic | Matched FACOMS | MOXIe Extension |
| --- | --- | --- | --- |
| Ethnicity (n [%]) | n | 136 | 136 |
|  | Hispanic or Latino | 6 (4.4%) | 6 (4.4%) |
|  | Not Hispanic or Latino | 129 (94.9%) | 130 (95.6%) |
|  | Not reported | 1 (0.7%) | 0 |
|  | p-value | - | 0.99 |
| Race (n [%]) | n | 130 | 136 |
|  | White | 125 (96.2%) | 133 (97.8%) |
|  | Non-White | 5 (3.8%) | 3 (2.2%) |
|  | p-value | - | 0.43 |
| Height (cm) | n | 89 | 136 |
|  | Mean (SD) | 165.1 (14.7) | 169.3 (10.4) |
|  | p-value | - | 0.020 |
| Weight (kg) | n | 95 | 136 |
|  | Mean (SD) | 61.0 (20.7) | 69.1 (16.7) |
|  | p-value | - | 0.0018 |
| BMI (kg/m^2^) | n | 89 | 136 |
|  | Mean (SD) | 22.0 (5.7) | 24.0 (5.2) |
|  | p-value | - | 0.0069 |
| Systolic Blood Pressure (mmHg) | n | 82 | 136 |
|  | Mean (SD) | 121.4 (15.0) | 121.1 (13.5) |
|  | p-value | - | 0.90 |
| Diastolic Blood Pressure (mmHg) | n | 82 | 136 |
|  | Mean (SD) | 73.2 (10.5) | 75.3 (8.7) |
|  | p-value | - | 0.15 |
| Heart Rate (beats/min) | n | 82 | 136 |
|  | Mean (SD) | 85.2 (15.4) | 79.8 (12.6) |
|  | p-value | - | 0.0089 |
| ADL Total Score | n | 124 | 136 |
|  | Mean (SD) | 11.8 (5.9) | 12.5 (4.9) |
|  | p-value | - | 0.28 |
| GAA1 Repeat Length | n | 129 | 119 |
|  | Mean (SD) | 590 (246) | 721 (270) |
|  | ≥ 675, n (%) | 54 (41.9%) | 66 (55.5%) |
|  | p-value | - | <0.0001 |
| GAA2 Repeat Length | n | 121 | 116 |
|  | Mean (SD) | 863 (232) | 728 (297) |
|  | p-value | - | 0.0001 |

Only patients with available information are summarized for each parameter. P-value for the difference between MOXIe Extension and Matched FACOMS was obtained by two-sample t test for Height, Weight, BMI, Systolic and Diastolic Blood Pressure, Heart Rate, ADL Total Score, GAA1 and GAA2 Repeat Length and by Chi-Square test for Ethnicity and Race.

Abbreviations: ADL=Activities of Daily Living; BMI=body mass index; SD=standard deviation

Supplementary Table 2: Demographics and Baseline Characteristics (FACOMS Patients Eligible for Matching who Were Not Matched to MOXIe Extension Patients in the Primary Pooled Population)

| Characteristic | Statistic | Non-Matched  FACOMS |
| --- | --- | --- |
| Age (years) | n | 462 |
|  | Mean (SD) | 22.4 (13.8) |
|  | Min, Max | 5, 73 |
| Age at FRDA Onset | n | 462 |
|  | Mean (SD) | 12.3 (8.6) |
| Sex (n [%]) | n | 462 |
|  | Female | 234 (50.6%) |
|  | Male | 228 (49.4%) |
| mFARS | n | 462 |
|  | Mean (SD) | 44.8 (18.1) |
|  | Min, Max | 2.0, 91.0 |
| Gait (Assessment #7 in FARS Section E [Upright Stability]) | n | 462 |
|  | Mean (SD) | 2.3 (1.69) |
| Ethnicity (n [%]) | n | 455 |
|  | Hispanic or Latino | 12 (2.6%) |
|  | Not Hispanic or Latino | 432 (94.9%) |
|  | Not reported | 11 (2.4%) |
| Race (n [%]) | n | 428 |
|  | White | 412 (96.3%) |
|  | Non-White | 16 (3.7%) |
| Height (cm) | n | 276 |
|  | Mean (SD) | 156.7 (19.2) |
| Weight (kg) | n | 299 |
|  | Mean (SD) | 52.4 (21.4) |
| BMI (kg/m^2^) | n | 270 |
|  | Mean (SD) | 20.2 (5.4) |
| Systolic Blood Pressure (mmHg) | n | 252 |
|  | Mean (SD) | 118.8 (14.2) |
| Diastolic Blood Pressure (mmHg) | n | 252 |
|  | Mean (SD) | 69.5 (9.1) |
| Heart Rate (beats/min) | n | 250 |
|  | Mean (SD) | 86.2 (14.7) |
| ADL Total Score | n | 432 |
|  | Mean (SD) | 11.6 (7.0) |
| GAA1 Repeat Length | n | 439 |
|  | Mean (SD) | 664 (225) |
|  | ≥ 675, n (%) | 233 (53.1%) |
| GAA2 Repeat Length | n | 426 |
|  | Mean (SD) | 942 (209) |

Abbreviations: ADL=Activities of Daily Living; BMI=body mass index; FRDA=Friedreich ataxia; FARS=Friedreich ataxia rating scale; Max=maximum; mFARS=modified Friedreich ataxia rating scale; Min=minimum; SD=standard deviation.

Supplementary Table 3: Demographics and Baseline Characteristics (Primary Placebo-Omav Population)

| Characteristic | Statistic | Matched FACOMS | MOXIe Extension |
| --- | --- | --- | --- |
| **Demographics and Baseline Characteristics Used as Covariates for Propensity Score Calculation** | | | |
| Age (years) | n | 95 | 95 |
|  | Mean (SD) | 28.4 (15.1) | 27.4 (7.5) |
|  | Min, Max | 6, 73 | 16, 41 |
|  | p-value | - | 0.54 |
| Age at FRDA Onset | n | 95 | 95 |
|  | Mean (SD) | 16.1 (11.6) | 15.5 (5.1) |
|  | p-value | - | 0.66 |
| Sex (n [%]) | n | 95 | 95 |
|  | Female | 43 (45.3%) | 43 (45.3%) |
|  | Male | 52 (54.7%) | 52 (54.7%) |
|  | p-value | - | 1 |
| mFARS | n | 95 | 95 |
|  | Mean (SD) | 44.5 (18.0) | 42.8 (12.8) |
|  | Min, Max | 8.5, 81.5 | 14.0, 73.5 |
|  | p-value | - | 0.45 |
| Gait (Assessment #7 in FARS Section E [Upright Stability]) | n | 95 | 95 |
|  | Mean (SD) | 3.0 (1.77) | 2.9 (1.36) |
|  | p-value | - | 0.58 |
| Other Demographics and Baseline Characteristics | | | |
| Ethnicity (n [%]) | n | 93 | 95 |
|  | Hispanic or Latino | 5 (5.4%) | 5 (5.3%) |
|  | Not Hispanic or Latino | 86 (92.5%) | 90 (94.7%) |
|  | Not reported | 2 (2.2%) | 0 |
|  | p-value | - | 0.94 |
| Race (n [%]) | n | 86 | 95 |
|  | White | 83 (96.5%) | 93 (97.9%) |
|  | Non-White | 3 (3.5%) | 2 (2.1%) |
|  | p-value | - | 0.57 |
| Height (cm) | n | 55 | 95 |
|  | Mean (SD) | 164.6 (15.1) | 169.6 (10.3) |
|  | p-value | - | 0.031 |
| Weight (kg) | n | 62 | 95 |
|  | Mean (SD) | 63.0 (23.5) | 69.7 (16.1) |
|  | p-value | - | 0.049 |
| BMI (kg/m^2^) | n | 55 | 95 |
|  | Mean (SD) | 22.9 (6.3) | 24.2 (5.1) |
|  | p-value | - | 0.18 |
| Systolic Blood Pressure (mmHg) | n | 50 | 95 |
|  | Mean (SD) | 122.1 (12.9) | 123.9 (12.5) |
|  | p-value | - | 0.40 |
| Diastolic Blood Pressure (mmHg) | n | 50 | 95 |
|  | Mean (SD) | 72.8 (9.6) | 76.9 (8.5) |
|  | p-value | - | 0.0097 |
| Heart Rate (beats/min) | n | 50 | 95 |
|  | Mean (SD) | 83.0 (13.6) | 79.6 (11.9) |
|  | p-value | - | 0.12 |
| ADL Total Score | n | 87 | 95 |
|  | Mean (SD) | 12.9 (6.8) | 12.8 (4.8) |
|  | p-value | - | 0.90 |
| GAA1 Repeat Length | n | 89 | 86 |
|  | Mean (SD) | 618 (246) | 717 (289) |
|  | ≥ 675, n (%) | 39 (43.8%) | 45 (52.3%) |
|  | p-value | - | 0.015 |
| GAA2 Repeat Length | n | 87 | 84 |
|  | Mean (SD) | 883 (223) | 721 (294) |
|  | p-value | - | <0.0001 |

Only patients with available information are summarized for each parameter. P-value for the difference between MOXIe Extension and Matched FACOMS was obtained by two-sample t test for Age, Age at FRDA Onset, mFARS, Gait, Height, Weight, BMI, Systolic and Diastolic Blood Pressure, Heart Rate, ADL Total Score, GAA1 and GAA2 Repeat Length and by Chi-Square test for Sex, Ethnicity and Race.

Abbreviations: ADL=Activities of Daily Living; BMI=body mass index; FRDA=Friedreich ataxia; FARS=Friedreich ataxia rating scale; Max=maximum; mFARS=modified Friedreich ataxia rating scale; Min=minimum; SD=standard deviation

**Supplementary Table 4: Demographics and Baseline Characteristics (Primary Omav-Omav Population)**

| Characteristic | Statistic | Matched FACOMS | MOXIe Extension |
| --- | --- | --- | --- |
| **Demographics and Baseline Characteristics Used as Covariates for Propensity Score Calculation** | | | |
| Age (years) | n | 41 | 41 |
|  | Mean (SD) | 26.3 (14.4) | 24.8 (6.3) |
|  | Min, Max | 7, 65 | 17, 40 |
|  | p-value | - | 0.54 |
| Age at FRDA Onset | n | 41 | 41 |
|  | Mean (SD) | 15.6 (8.9) | 15.3 (5.7) |
|  | p-value | - | 0.85 |
| Sex (n [%]) | n | 41 | 41 |
|  | Female | 27 (65.9%) | 27 (65.9%) |
|  | Male | 14 (34.1%) | 14 (34.1%) |
|  | p-value | - | 1 |
| mFARS | n | 41 | 41 |
|  | Mean (SD) | 39.6 (16.8) | 40.9 (12.2) |
|  | Min, Max | 13.0, 71.5 | 8.2, 62.0 |
|  | p-value | - | 0.71 |
| Gait (Assessment #7 in FARS Section E [Upright Stability]) | n | 41 | 41 |
|  | Mean (SD) | 2.6 (1.62) | 2.7 (1.37) |
|  | p-value | - | 0.88 |
| Other Demographics and Baseline Characteristics | | | |
| Ethnicity (n [%]) | n | 41 | 41 |
|  | Hispanic or Latino | 1 (2.4%) | 1 (2.4%) |
|  | Not Hispanic or Latino | 39 (95.1%) | 40 (97.6%) |
|  | Not reported | 1 (2.4%) | 0 |
|  | p-value | - | 0.99 |
| Race (n [%]) | n | 38 | 41 |
|  | White | 37 (97.4%) | 40 (97.6%) |
|  | Non-White | 1 (2.6%) | 1 (2.4%) |
|  | p-value | - | 0.96 |
| Height (cm) | n | 27 | 41 |
|  | Mean (SD) | 159.4 (14.7) | 168.6 (10.9) |
|  | p-value | - | 0.0042 |
| Weight (kg) | n | 29 | 41 |
|  | Mean (SD) | 59.1 (20.9) | 67.6 (18.3) |
|  | p-value | - | 0.077 |
| BMI (kg/m^2^) | n | 27 | 41 |
|  | Mean (SD) | 22.8 (6.0) | 23.6 (5.5) |
|  | p-value | - | 0.58 |
| Systolic Blood Pressure  (mmHg) | n | 28 | 41 |
|  | Mean (SD) | 120.6 (14.7) | 114.6 (13.7) |
|  | p-value | - | 0.087 |
| Diastolic Blood Pressure (mmHg) | n | 28 | 41 |
|  | Mean (SD) | 72.2 (9.6) | 71.5 (7.9) |
|  | p-value | - | 0.75 |
| Heart Rate (beats/min) | n | 27 | 41 |
|  | Mean (SD) | 82.2 (15.1) | 80.4 (14.3) |
|  | p-value | - | 0.62 |
| ADL Total Score | n | 37 | 41 |
|  | Mean (SD) | 10.8 (5.9) | 11.9 (5.2) |
|  | p-value | - | 0.39 |
| GAA1 Repeat Length | n | 39 | 33 |
|  | Mean (SD) | 591 (240) | 732 (216) |
|  | ≥ 675, n (%) | 19 (48.7%) | 21 (63.6%) |
|  | p-value | - | 0.011 |
| GAA2 Repeat Length | n | 37 | 32 |
|  | Mean (SD) | 880 (239) | 746 (308) |
|  | p-value | - | 0.046 |

Only patients with available information are summarized for each parameter. P-value for the difference between MOXIe Extension and Matched FACOMS was obtained by two-sample t test for Age, Age at FRDA Onset, mFARS, Gait, Height, Weight, BMI, Systolic and Diastolic Blood Pressure, Heart Rate, ADL Total Score, GAA1 and GAA2 Repeat Length and by Chi-Square test for Sex, Ethnicity and Race.

Abbreviations: ADL=Activities of Daily Living; BMI=body mass index; FRDA=Friedreich ataxia; FARS=Friedreich ataxia rating scale; Max=maximum; mFARS=modified Friedreich ataxia rating scale; Min=minimum; SD=standard deviation

Supplementary Table 5: Demographics and Baseline Characteristics (Sensitivity Pooled Population)

| Characteristic | Statistic | Matched FACOMS | MOXIe Extension |
| --- | --- | --- | --- |
| **Demographics and Baseline Characteristics Used as Covariates for Propensity Score Calculation** | | | |
| Age (years) | n | 133 | 133 |
|  | Mean (SD) | 26.5 (7.7) | 26.3 (7.1) |
|  | Min, Max | 16, 41 | 16, 41 |
|  | p-value | - | 0.85 |
| Age at FRDA Onset | n | 133 | 133 |
|  | Mean (SD) | 15.2 (5.5) | 15.2 (4.9) |
|  | p-value | - | 0.96 |
| Sex (n [%]) | n | 133 | 133 |
|  | Female | 67 (50.4%) | 67 (50.4%) |
|  | Male | 66 (49.6%) | 66 (49.6%) |
|  | p-value | - | 1 |
| mFARS | n | 133 | 133 |
|  | Mean (SD) | 43.8 (16.4) | 42.5 (12.6) |
|  | Min, Max | 11.0, 73.0 | 8.2, 73.5 |
|  | p-value | - | 0.48 |
| Gait (Assessment #7 in FARS Section E [Upright Stability]) | n | 133 | 133 |
|  | Mean (SD) | 2.9 (1.69) | 2.8 (1.37) |
|  | p-value | - | 0.78 |
| Other Demographics and Baseline Characteristics | | | |
| Ethnicity (n [%]) | n | 132 | 133 |
|  | Hispanic or Latino | 2 (1.5%) | 5 (3.8%) |
|  | Not Hispanic or Latino | 126 (95.5%) | 128 (96.2%) |
|  | Not reported | 4 (3.0%) | 0 |
|  | p-value | - | 0.27 |
| Race (n [%]) | n | 126 | 133 |
|  | White | 122 (96.8%) | 130 (97.7%) |
|  | Non-White | 4 (3.2%) | 3 (2.3%) |
|  | p-value | - | 0.65 |
| Height (cm) | n | 77 | 133 |
|  | Mean (SD) | 171.0 (9.9) | 169.3 (10.5) |
|  | p-value | - | 0.26 |
| Weight (kg) | n | 85 | 133 |
|  | Mean (SD) | 66.8 (14.1) | 68.8 (16.7) |
|  | p-value | - | 0.36 |
| BMI (kg/m^2^) | n | 75 | 133 |
|  | Mean (SD) | 22.9 (4.3) | 23.9 (5.2) |
|  | p-value | - | 0.14 |
| Systolic Blood Pressure  (mmHg) | n | 66 | 133 |
|  | Mean (SD) | 123.4 (11.8) | 121.1 (13.7) |
|  | p-value | - | 0.24 |
| Diastolic Blood Pressure (mmHg) | n | 66 | 133 |
|  | Mean (SD) | 73.5 (8.9) | 75.2 (8.7) |
|  | p-value | - | 0.22 |
| Heart Rate (beats/min) | n | 66 | 133 |
|  | Mean (SD) | 80.9 (14.9) | 79.9 (12.6) |
|  | p-value | - | 0.61 |
| ADL Total Score | n | 125 | 133 |
|  | Mean (SD) | 12.3 (6.3) | 12.6 (4.9) |
|  | p-value | - | 0.77 |
| GAA1 Repeat Length | n | 127 | 116 |
|  | Mean (SD) | 574 (198) | 721 (271) |
|  | ≥ 675, n (%) | 41 (32.3%) | 64 (55.2%) |
|  | p-value | - | <0.0001 |
| GAA2 Repeat Length | n | 122 | 113 |
|  | Mean (SD) | 870 (245) | 739 (290) |
|  | p-value | - | 0.0002 |

Only patients with available information are summarized for each parameter. P-value for the difference between MOXIe Extension and Matched FACOMS was obtained by two-sample t test for Age, Age at FRDA Onset, mFARS, Gait, Height, Weight, BMI, Systolic and Diastolic Blood Pressure, Heart Rate, ADL Total Score, GAA1 and GAA2 Repeat Length and by Chi-Square test for Sex, Ethnicity and Race.

Abbreviations: ADL=Activities of Daily Living; BMI=body mass index; FRDA=Friedreich ataxia; FARS=Friedreich ataxia rating scale; Max=maximum; mFARS=modified Friedreich ataxia rating scale; Min=minimum; SD=standard deviation

Supplementary Table 6: Demographics and Baseline Characteristics (Sensitivity Placebo-Omav Population)

| Characteristic | Statistic | Matched FACOMS | MOXIe Extension |
| --- | --- | --- | --- |
| **Demographics and Baseline Characteristics Used as Covariates for Propensity Score Calculation** | | | |
| Age (years) | n | 95 | 95 |
|  | Mean (SD) | 27.2 (7.4) | 27.4 (7.5) |
|  | Min, Max | 16, 41 | 16, 41 |
|  | p-value | - | 0.88 |
| Age at FRDA Onset | n | 95 | 95 |
|  | Mean (SD) | 15.6 (5.9) | 15.5 (5.1) |
|  | p-value | - | 0.92 |
| Sex (n [%]) | n | 95 | 95 |
|  | Female | 43 (45.3%) | 43 (45.3%) |
|  | Male | 52 (54.7%) | 52 (54.7%) |
|  | p-value | - | 1 |
| mFARS | n | 95 | 95 |
|  | Mean (SD) | 43.4 (15.6) | 42.8 (12.8) |
|  | Min, Max | 13.0, 73.0 | 14.0, 73.5 |
|  | p-value | - | 0.79 |
| Gait (Assessment #7 in FARS Section E [Upright Stability]) | n | 95 | 95 |
|  | Mean (SD) | 2.9 (1.64) | 2.9 (1.36) |
|  | p-value | - | 0.92 |
| Other Demographics and Baseline Characteristics | | | |
| Ethnicity (n [%]) | n | 94 | 95 |
|  | Hispanic or Latino | 3 (3.2%) | 5 (5.3%) |
|  | Not Hispanic or Latino | 90 (95.7%) | 90 (94.7%) |
|  | Not reported | 1 (1.1%) | 0 |
|  | p-value | - | 0.49 |
| Race (n [%]) | n | 90 | 95 |
|  | White | 88 (97.8%) | 93 (97.9%) |
|  | Non-White | 2 (2.2%) | 2 (2.1%) |
|  | p-value | - | 0.96 |
| Height (cm) | n | 53 | 95 |
|  | Mean (SD) | 172.0 (9.4) | 169.6 (10.3) |
|  | p-value | - | 0.17 |
| Weight (kg) | n | 61 | 95 |
|  | Mean (SD) | 68.8 (14.9) | 69.7 (16.1) |
|  | p-value | - | 0.72 |
| BMI (kg/m^2^) | n | 53 | 95 |
|  | Mean (SD) | 23.2 (5.4) | 24.2 (5.1) |
|  | p-value | - | 0.25 |
| Systolic Blood Pressure  (mmHg) | n | 48 | 95 |
|  | Mean (SD) | 125.8 (10.3) | 123.9 (12.5) |
|  | p-value | - | 0.39 |
| Diastolic Blood Pressure (mmHg) | n | 48 | 95 |
|  | Mean (SD) | 74.0 (8.4) | 76.9 (8.5) |
|  | p-value | - | 0.061 |
| Heart Rate (beats/min) | n | 48 | 95 |
|  | Mean (SD) | 80.9 (12.2) | 79.6 (11.9) |
|  | p-value | - | 0.55 |
| ADL Total Score | n | 87 | 95 |
|  | Mean (SD) | 12.0 (5.6) | 12.8 (4.8) |
|  | p-value | - | 0.31 |
| GAA1 Repeat Length | n | 91 | 86 |
|  | Mean (SD) | 573 (208) | 717 (289) |
|  | ≥ 675, n (%) | 30 (33.0%) | 45 (52.3%) |
|  | p-value | - | 0.0002 |
| GAA2 Repeat Length | n | 87 | 84 |
|  | Mean (SD) | 881 (232) | 721 (294) |
|  | p-value | - | 0.0001 |

Only patients with available information are summarized for each parameter. P-value for the difference between MOXIe Extension and Matched FACOMS was obtained by two-sample t test for Age, Age at FRDA Onset, mFARS, Gait, Height, Weight, BMI, Systolic and Diastolic Blood Pressure, Heart Rate, ADL Total Score, GAA1 and GAA2 Repeat Length and by Chi-Square test for Sex, Ethnicity and Race.

Abbreviations: ADL=Activities of Daily Living; BMI=body mass index; FRDA=Friedreich ataxia; FARS=Friedreich ataxia rating scale; Max=maximum; mFARS=modified Friedreich ataxia rating scale; Min=minimum; SD=standard deviation

Supplementary Table 7: Demographics and Baseline Characteristics (Sensitivity Omav-Omav Population)

| Characteristic | Statistic | Matched FACOMS | MOXIe Extension |
| --- | --- | --- | --- |
| **Demographics and Baseline Characteristics Used as Covariates for Propensity Score Calculation** | | | |
| Age (years) | n | 40 | 40 |
|  | Mean (SD) | 24.5 (6.8) | 24.5 (6.1) |
|  | Min, Max | 16, 40 | 17, 40 |
|  | p-value | - | 0.99 |
| Age at FRDA Onset | n | 40 | 40 |
|  | Mean (SD) | 14.9 (4.7) | 14.8 (4.7) |
|  | p-value | - | 0.92 |
| Sex (n [%]) | n | 40 | 40 |
|  | Female | 26 (65.0%) | 26 (65.0%) |
|  | Male | 14 (35.0%) | 14 (35.0%) |
|  | p-value | - | 1 |
| mFARS | n | 40 | 40 |
|  | Mean (SD) | 42.6 (16.3) | 41.2 (12.2) |
|  | Min, Max | 13.0, 72.0 | 8.2, 62.0 |
|  | p-value | - | 0.66 |
| Gait (Assessment #7 in FARS Section E [Upright Stability]) | n | 40 | 40 |
|  | Mean (SD) | 2.8 (1.71) | 2.7 (1.38) |
|  | p-value | - | 0.77 |
| Other Demographics and Baseline Characteristics | | | |
| Ethnicity (n [%]) | n | 38 | 40 |
|  | Hispanic or Latino | 2 (5.3%) | 1 (2.5%) |
|  | Not Hispanic or Latino | 35 (92.1%) | 39 (97.5%) |
|  | Not reported | 1 (2.6%) | 0 |
|  | p-value | - | 0.51 |
| Race (n [%]) | n | 35 | 40 |
|  | White | 35 (100%) | 39 (97.5%) |
|  | Non-White | 0 | 1 (2.5%) |
|  | p-value | - | 0.35 |
| Height (cm) | n | 27 | 40 |
|  | Mean (SD) | 169.4 (9.3) | 168.4 (10.9) |
|  | p-value | - | 0.70 |
| Weight (kg) | n | 27 | 40 |
|  | Mean (SD) | 61.8 (14.5) | 66.9 (18.1) |
|  | p-value | - | 0.23 |
| BMI (kg/m^2^) | n | 27 | 40 |
|  | Mean (SD) | 21.6 (5.3) | 23.4 (5.5) |
|  | p-value | - | 0.18 |
| Systolic Blood Pressure  (mmHg) | n | 23 | 40 |
|  | Mean (SD) | 121.3 (12.5) | 114.2 (13.6) |
|  | p-value | - | 0.046 |
| Diastolic Blood Pressure (mmHg) | n | 23 | 40 |
|  | Mean (SD) | 73.9 (9.6) | 71.2 (7.6) |
|  | p-value | - | 0.22 |
| Heart Rate (beats/min) | n | 23 | 40 |
|  | Mean (SD) | 80.7 (15.7) | 80.7 (14.3) |
|  | p-value | - | 0.99 |
| ADL Total Score | n | 38 | 40 |
|  | Mean (SD) | 11.5 (6.4) | 12.1 (5.1) |
|  | p-value | - | 0.60 |
| GAA1 Repeat Length | n | 40 | 32 |
|  | Mean (SD) | 585 (201) | 726 (216) |
|  | ≥ 675, n (%) | 17 (42.5%) | 20 (62.5%) |
|  | p-value | - | 0.0057 |
| GAA2 Repeat Length | n | 39 | 31 |
|  | Mean (SD) | 872 (246) | 758 (305) |
|  | p-value | - | 0.088 |

Only patients with available information are summarized for each parameter. P-value for the difference between MOXIe Extension and Matched FACOMS was obtained by two-sample t test for Age, Age at FRDA Onset, mFARS, Gait, Height, Weight, BMI, Systolic and Diastolic Blood Pressure, Heart Rate, ADL Total Score, GAA1 and GAA2 Repeat Length and by Chi-Square test for Sex, Ethnicity and Race.

Abbreviations: ADL=Activities of Daily Living; BMI=body mass index; FRDA=Friedreich ataxia; FARS=Friedreich ataxia rating scale; Max=maximum; mFARS=modified Friedreich ataxia rating scale; Min=minimum; SD=standard deviation

**Supplementary Table 8**: **Propensity Score Diagnostic Results (Sensitivity Populations)**

| Diagnostic | Criteria  **for Good or Acceptable Match**^a^ | Pooled  (Match 4) | Placebo-Omav  (Match 5) | Omav-Omav  (Match 6) |
| --- | --- | --- | --- | --- |
|  |  | Score | Score | Score |
| Standardized Difference of the Means of the Propensity Score | <0.5 | 0.0441 | 0.0538 | 0.0065 |
| Standardized Difference of the Means of Covariates | | | | |
| Sex | <0.5 | 0 | 0 | 0 |
| Baseline Gait | <0.5 | 0.0343 | 0.0140 | 0.0643 |
| Baseline mFARS | <0.5 | 0.0872 | 0.0380 | 0.0995 |
| Age at baseline | <0.5 | 0.0234 | 0.0225 | 0.0039 |
| Age at FRDA Onset | <0.5 | 0.0058 | 0.0152 | 0.0212 |
| Ratio of the Variances of the Propensity Score | Close to 1; >0.8 and <1.25 | 1.10 | 1.18 | 1.00 |
| Ratio of the Variances of the Residuals for Covariates | | | | |
| Sex | 0.5 to 2 | 0.998 | 0.998 | 0.994 |
| Baseline Gait | 0.5 to 2 | 0.637 | 0.672 | 0.648 |
| Baseline mFARS | 0.5 to 2 | 0.570 | 0.653 | 0.694 |
| Age at baseline | 0.5 to 2 | 0.718 | 0.775 | 0.818 |
| Age at FRDA Onset | 0.5 to 2 | 0.811 | 0.751 | 1.18 |

^a^Criteria for a “good” match shown for standardized difference of the means of the propensity score, standardized difference of the means of the propensity score for each covariate, and ratio of the variances of the propensity score. Criteria for an “acceptable” match shown for the ratio of the variances of the residuals for each covariate.

Abbreviations: FRDA=Friedreich ataxia; mFARS=modified Friedreich ataxia rating scale

Supplementary Table 9: Change in mFARS over 3 years (FACOMS Patients Eligible for Matching who Were Not Matched to MOXIe Extension Patients in the Primary Pooled Population)

|  | Baseline | | mFARS Change from Baseline | | | | | |
| --- | --- | --- | --- | --- | --- | --- | --- | --- |
|  |  |  | Year 1 | | Year 2 | | Year 3 | |
|  | N | Mean (SD) | N | LS Mean (±SE) | N | LS Mean (±SE) | N | LS Mean  (±SE) |
| Non-Matched  FACOMS | 462 | 44.8 (18.1) | 392 | 2.22 (0.33) | 327 | 3.54 (0.35) | 276 | 5.72 (0.37) |

Abbreviations: LS=least squares; mFARS=modified Friedreich ataxia rating scale; SD=standard deviation; SE=standard error

**Supplementary Table 10: mFARS Change in Sensitivity Populations**

| Analysis Population | mFARS Change from Baseline (LS Mean [±SE]) | | |
| --- | --- | --- | --- |
|  | **Year 1** | **Year 2** | **Year 3** |
| Sensitivity Pooled (Match 4)  Difference | -1.50 (0.71)  p=0.035 | -1.57 (0.74)  p=0.035 | -2.39 (0.82)  p=0.0039 |
| Sensitivity Placebo-Omav (Match 5)  Difference | -2.39 (0.88)  p=0.0072 | -2.09 (0.92)  p=0.024 | -3.15 (1.00)  p=0.0018 |
| Sensitivity Omav-Omav (Match 6)  Difference | -1.14 (1.33)  p=0.40 | -3.41 (1.35)  p=0.013 | -4.68 (1.55)  p=0.0030 |

Difference is MOXIe Extension – matched FACOMS.

Abbreviations: LS=least squares; mFARS=modified Friedreich ataxia rating scale; SE=standard error

Note: Each Sensitivity population (Pooled, Placebo-Omav, Omav-Omav) represents a different set of matched patients (Match 4, Match 5, or Match 6).

Supplementary Table 11: mFARS Study Day at Each Analysis Visit

|  | Total mFARS Study Day | | | | | |
| --- | --- | --- | --- | --- | --- | --- |
|  | Year 1 | | Year 2 | | Year 3 | |
|  | **Matched**  FACOMS | MOXIe Extension | **Matched**  FACOMS | MOXIe Extension | **Matched**  FACOMS | MOXIe Extension |
| **Primary Pooled Population** | | | | | | |
| N | 108 | 133 | 103 | 102 | 83 | 77 |
| Mean  (SD) | 373.0 (60.89) | 343.3 (79.85) | 755.1 (70.31) | 724.2 (79.50) | 1125.8 (78.30) | 1029.8 (49.79) |
| **Primary Placebo-Omav Population** | | | | | | |
| N | 72 | 95 | 71 | 69 | 64 | 56 |
| Mean  (SD) | 374.3 (66.72) | 336.8 (78.80) | 730.4 (82.20) | 727.0 (80.63) | 1101.1 (83.54) | 1026.7 (44.25) |
| **Primary Omav-Omav Population** | | | | | | |
| N | 34 | 38 | 33 | 33 | 25 | 21 |
| Mean  (SD) | 368.7 (61.22) | 359.5 (81.20) | 745.0 (71.24) | 718.2 (77.96) | 1112.6 (67.29) | 1038.0 (62.76) |
| **Sensitivity Pooled Population** | | | | | | |
| N | 102 | 130 | 93 | 99 | 82 | 74 |
| Mean  (SD) | 377.1 (68.87) | 343.6 (80.75) | 756.9 (78.39) | 725.9 (80.03) | 1112.6 (85.39) | 1030.8 (50.56) |
| **Sensitivity Placebo-Omav Population** | | | | | | |
| N | 71 | 95 | 72 | 69 | 60 | 56 |
| Mean  (SD) | 380.8 (63.26) | 336.8 (78.80) | 756.0 (76.89) | 727.0 (80.63) | 1127.5 (80.69) | 1026.7 (44.25) |
| **Sensitivity Omav-Omav Population** | | | | | | |
| N | 30 | 37 | 29 | 32 | 24 | 20 |
| Mean  (SD) | 403.0 (59.25) | 360.1 (82.22) | 764.9 (88.23) | 720.0 (78.44) | 1117.9 (77.82) | 1039.9 (63.80) |

Abbreviations: mFARS=modified Friedreich ataxia rating scale; SD=standard deviation

SUPPLEMENTARY STATISTICAL APPENDIX

The logistic regression modeling, matching, and assessment of the quality of the matches were executed using PROC PSMATCH in SAS with additional diagnostics computed separately. The following options within PSMATCH were used:

- The region used was that of the common support (REGION = CS).
- Optimal matching was used to create the matches.
- Matching for sex was exact, i.e., females were only matched to females and males were only matched to males.
- The distance between matches was measured on the logit of the propensity score.
- The caliper (the specified distance between observations for declaring a “match”) for matching was set to missing and no weighting was used.
- The assessment for the quality of matches was conducted for the logit of the propensity score, the propensity score, and all covariates in the model.

The estimated propensity score $\hat{e}_{i}$ for the *i*^th^ patient was computed as follows:

$$\hat{e}_{i}= \frac{exp\left( \hat{\beta}x_{i} \right)}{1+exp\left( \hat{\beta}x_{i} \right)} ,$$

where $\hat{\beta}$ denotes the estimate of $\beta$ obtained from a logistic model, where the probability that a patient is a member of the treated group given a covariate vector $x$, written as P(Y=1|$x$), was defined as follows:

$$\hat{e}_{i}= P\left( Y=1| x_{i} \right)= \frac{exp\left( \hat{\beta}x_{i} \right)}{1+exp\left( \hat{\beta}x_{i} \right).}$$

Note that $\hat{e}_{i}$ is a probability with 0 ≤ $\hat{e}_{i}$ ≤ 1. The analysis used the estimated linear propensity score $g_{i}$, which was calculated as $g_{i}$ = $\hat{\beta}x_{i}.$
